# Effects of Dissolved Organic Matter on Uptake and Translocation of Lead in *Brassica* *chinensis* and Potential Health Risk of Pb

**DOI:** 10.3390/ijerph13070687

**Published:** 2016-07-08

**Authors:** Renying Li, Zhigao Zhou, Xiaojin Xie, Yingxue Li, Yaohong Zhang, Xianghua Xu

**Affiliations:** 1 Jiangsu Key Laboratory of Agricultural Meteorology, Nanjing University of Information Science & Technology, Nanjing 210044, China; xxj_200210@nuist.edu.cn (X.X.); lyxue@nuist.edu.cn (Y.L.); yhzhang@nuist.edu.cn (Y.Z.); xianghua_xu@163.com (X.X.); 2 Key Laboratory of Soil Environment and Pollution Remediation, Institute of Soil Science, Chinese Academy of Sciences, Nanjing 210008, China; zgzhou@issas.ac.cn; 3 School of Chemical Engineering and Energy, Zhengzhou University, Zhengzhou 450001, China

**Keywords:** *Brassica chinensis*, dissolved organic matter, lead, uptake, translocation

## Abstract

Dissolved organic matter (DOM) can affect the bioavailability of heavy metals in soil, especially in soils used for vegetable production, where intensive organic fertilization is applied. The present study examined the effects of DOM derived from commercial organic fertilizers (COF), cow manure (COM) and chicken manure (CHM), on uptake and translocation of lead (Pb) in *Brassica chinensis* in a pot experiment. The results indicate that DOM derived from CHM (DOM_CHM_) significantly increased Pb concentrations in roots of *B. chinensis* (*p* < 0.05). By contrast, there was no significant increase in shoot Pb concentration for all the DOM treatments except the high DOM_CHM_ treatment in the soil with 800 mg·kg^−1^ Pb. Consistent with the Pb concentrations in shoots, translocation factor of Pb from soil to shoot and specific lead uptake (SLU) by *B.* *chinensis* were significantly increased for the high DOM_CHM_ treatment in the high Pb soil, but not for other DOM treatments. Based on the results of this study, the application of DOM to the soil with 800 mg·kg^−1^ Pb could result in an increase in total Pb annually ingested by the inhabitants of Nanjing City in the range of 2018–9640 kg, with the highest estimates resulting from the high DOM_CHM_ treatment. This study suggests the risk may rise under some conditions as indicated in the high DOM_CHM_ treatment and high Pb pollution level.

## 1. Introduction

Pollution of lead (Pb) has raised a great concern on the health of human and other organisms because of its toxicity and persistence in the environment. Previous studies have shown that Pb can induce various morphological, physiological and biochemical abnormalities in plants such as decrease in seed germination, plant growth and chlorophyll production, and upsets in mineral nutrition and water balance [1,2,3].

The bioavailability of Pb to plants is closely related to its chemical speciation in soil [4,5,6]. Dissolved organic matter (DOM), through forming complexes with Pb, plays a significant role in Pb speciation in soil and thus its availability to plants [7,8,9]. Khan et al. [9] observed that Pb and cadmium (Cd) concentrations in wheat were increased when humic acid-rich sewage was irrigated, and concluded that humic acids enhanced the availability of Pb and Cd to plants.

Domestic animal manure is an important source of organic fertilizer for agricultural soils, particularly in intensive vegetable production areas, because of its ability to enhance vegetable growth and production through providing nutrients and improving soil structure [10,11]. On the other hand, animal manure application can increase DOM concentration in soils, which may escalate the risk of heavy metal pollution due to its positive effect on solubility and mobility (i.e., bioavailability) of heavy metals in soils [7,9]. DOM is a complex mixture, and its composition, concentration and then effect on bioavailability of heavy metals may depend largely on its source [12,13]. Cow and chicken manures are two of the main forms of organic fertilizers applied to the soils used to grow vegetables [14]. Commercial organic matter is also widely applied to soils under various cropping systems including vegetable growing [15]. Traditionally, these organic fertilizers are applied at rates based on their N (or P) content and prescribed percentage to total N (or P) input as fertilizers, and usually at larger rates for vegetable-growing soils than for grain crops-growing soils [16]. However, there is little information on the risk of enhanced DOM due to higher application rates of organic fertilizers in vegetable-growing soils, which are lightly and moderately polluted by Pb. In practice, these organic fertilizers are applied to vegetable soils based on their prescribed N or P contribution. Considering their significant difference in DOM release from similar application rates, it is important to understand the effects of DOM derived from these widely-used organic fertilizers at concentrations likely occurring in the field on Pb accumulation in vegetables.

Soil-to-plant translocation factor is an index for evaluating the translocation potential of a metal from soil to plant [17]. The translocation factor is generally defined as the ratio of the metal concentration in the plant to the total metal concentration in the soil [17]. Some studies have indicated that the translocation factor of heavy metals depends on plant species [17,18], heavy metal concentration in soil [19] and environmental conditions [20]. As mentioned above, DOM can affect soil Pb availability to plants. It is still unclear how the translocation factor of Pb may vary with DOM source and concentration in vegetable-grown soils.

In the present study, we used *Brassica chinensis* in pot experiment because it is one of the primary vegetables consumed in China. The objectives of this study were to: (1) investigate the effect of DOM of three widely-used organic fertilizers at different concentrations likely occurring in vegetable-grown soils on Pb accumulation and uptake in *B. chinensis*; and (2) assess the effect of DOM of various sources at different concentrations on the translocation of Pb from soil to *B. chinensis*. In addition, we discussed the potential risk of elevated Pb concentration in *B. chinensis* due to enhanced DOM in vegetable-grown soils as suggested in this study.

## 2. Materials and Methods

### 2.1. Soil and DOM Preparation

The soil used in this study was collected from a suburb area of the city of Nanjing, Jiangsu Province, China. After being transported to the laboratory, the soil was air-dried, sieved (<5 mm) and homogenized for a pot experiment. Subsamples were ground through a 10 (<2 mm) and 100 (<0.149 mm) mesh sieves for analyzing its chemical and physical properties. Selected soil properties were analyzed according to the methods recommended by the Soil Science Society of China [21]. Soil pH was measured by pH meter using a 1:2.5 soil to water ratio. Organic matter content was determined by the wet combustion method [22]. Total nitrogen (N) was determined by the Kjeldahl method. Total phosphorus (P) and total potassium (K) were measured by the colorimetric molybdenum blue method and flame photometer (Shangyi 6400A), respectively, after the soil subsample (<0.149 mm) was digested by the three-acid (HF-HClO_4_-HNO_3_) method. Total Pb, Cd, Cr, Cu and Zn were measured by ICP-AES (Optima 8000) using the same digest. Clay content was determined using a laser particle size analyzer (LS 13320). The pH of the soil was 6.81. The soil contained 14.0 mg·g^−1^ organic matter, 0.92 mg·g^−1^ total N, 0.58 mg·g^−1^ total P, 17.4 mg·g^−1^ total K, and 15.2% clay (*v*/*v*). Total Pb, Cd, Cr, Cu and Zn concentrations in the soil were 7.15, 1.40, 33.33, 13.66 and 69.51 mg·kg^−1^, respectively.

Samples of commercial organic fertilizer (COF), cow manure (COM) and chicken manure (CHM) were collected from a commercial farm in the suburb of Nanjing. The organic matter samples were air-dried, ground and sieved (<0.5 mm). DOM samples were prepared using a modified method reported by Barricuso et al. [23]. Specifically, each of the organic matter samples was first shaken for 8 h at an organic matter-to-water ratio of 1:20 (*w*/*v*), then incubated aerobically for one week at 25 °C, and, finally, centrifuged for 30 min (4500 r/min) and filtered through a 0.45 μm membrane. The resulting filtrates were considered as the DOM samples of various sources, which were used in this study. The pH of the DOM samples was measured by pH meter. Their total organic carbon (TOC) concentrations were determined using a liquid C/N analyzer (Vario Toc). Total Pb, Cd, Cr, Cu and Zn concentrations of the DOM samples were determined by ICP-AES (Optima 8000). The basic physicochemical properties of the DOM samples are shown in Table 1.

### 2.2. Experimental Setup

A pot experiment was conducted in a greenhouse with a temperature ranging between 20 and 30 °C in a day. Three levels of Pb addition: 0 (Pb0), 100 (Pb100) and 800 mg Pb·kg^−1^ soil (Pb800), and three DOM samples prepared from COF, COM and CHM (named as DOM_COF_, DOM_COM_ and DOM_CHM_) each with two dosages (low: 100 mL and high: 500 mL) were used in the pot experiment. The DOM-free treatment (without addition of DOM) at each Pb level was the common blank control for all the DOM treatments at the same soil Pb level. Each treatment was replicated three times. For each Pb-added treatment (pot), a required aliquot of Pb solution (as Pb(NO_3_)_2_) was added to 1.2 kg of soil, mixed thoroughly, watered to 60% field water holding capacity and left at room temperature for two weeks. In addition to spiked Pb, basic fertilizers including 150 mg·N·kg^−1^ soil as (NH_4_)_2_SO_4_, 100 mg·P_2_O_5_·kg^−1^ soil as KH_2_PO_4_, and 150 mg·K_2_O·kg^−1^ soil as K_2_SO_4_ and KH_2_PO_4_, were mixed thoroughly with the soil in each pot.

### 2.3. Plant Culture, Sample Collection and Analysis

Seeds of *B. chinensis* were soaked overnight in distilled water and then placed on a wetted filter paper at 30 °C in an incubator. After two days, four seeds were transplanted into each pot, which were thinned to two seedlings in each pot after coming out. After one month of growth, either 100 or 500 mL of DOM samples prepared from the three sources were added to the pots except the DOM-free controls.

During the growth of *B. chinensis*, distilled water was added and soil moisture was adjusted to 60% of its field water capacity by weighing the loaded pots. Fifty days after transplantation, the plants were harvested, separated into shoots and roots, rinsed with tap water and then distilled water, dried at 70 °C for 48 h, and weighed. Dried plant samples were ground and digested using HNO_3_ and HClO_4_ [11,21]. Pb in the digests was determined by ICP-AES (Optima 8000). A reagent blank and standard reference plant material (GBW07603 from the Chinese National Center for Standard Materials) were included in the analysis for the analytical quality control.

### 2.4. The Soil-to-Plant Translocation Factor of Pb and Specific Lead Uptake (SLU)

The ability of *B.* *chinensis* to translocate and take up Pb was assessed using the translocation factor (TF) [17] and specific lead uptake (SLU) [24] as follows,

$$TF=\frac{{\left[Pb\right]}_{shoot}}{{\left[Pb\right]}_{soil}}$$

$$SLU=\frac{{\left[Pb\right]}_{root}\times Biomass_{root}+{\left[Pb\right]}_{shoot}\times Biomass_{shoot}}{Biomass_{root}}$$

where [*Pb*]*_shoot_*, [*Pb*]*_root_* and [*Pb*]*_soil_* are the concentrations of Pb in shoots, roots and soil, respectively; and *Biomass_shoot_* and *Biomass_root_* are the dry weights of shoots and roots of *B.* *chinensis*, respectively.

### 2.5. Potential Health Risk of Pb

To estimate the potential health risk posed by the higher concentrations of Pb resulting from the addition of DOM to the soil, the increase of the amount of Pb ingested by the population was calculated using the following Equation (1), taking Nanjing City (Jiangsu Province, China) as an example, based on the data from the soil with 800 mg·kg^−1^ Pb added in the present study.

*I_a_= D_i_* × *C* × *E_t_* × *P* × *I_p_* (1)

*I_a_* is the increase of the amount of Pb ingested by the population (kg); *D_i_* is amount of *B. chinensis* (g·person^−1^·day^−1^) consumed; *C* is the Pb concentration in the edible part of *B. chinensis* grown in soil with 800 mg·kg^−1^Pb (7.42 mg·kg^−1^); *E_t_* is the exposure time (365 days); *P* is population (8.24 million) of Nanjing City in 2015; *I_p_* is the percent increase in Pb concentration in the edible parts of the vegetable when DOM is added to the soil (%), compared with DOM-free treatment.

### 2.6. Statistical Analysis

Data are expressed as means ± standard error (SE). The Data were analyzed with a one-way analysis of variance (ANOVA) approach, using SPSS software program (SPSS, Inc., Chicago version 19.0, Chicago, IL, USA). Differences among the treatments were tested using Least Significant Difference (LSD) test.

## 3. Results

### 3.1. Plant Growth

There was no significant difference among the biomass (shoots or roots) of *B. chinensis* grown in the soils at different Pb levels with no DOM addition (the controls at the first row in Table 2), indicating that the addition of 100 or 800 mg·kg^−1^ Pb to the soil had no toxic effect on the growth of *B.* *chinensis* (Table 2). The addition of DOM except the high DOM_CHM_ treatment in the Pb800 soil had no significant effect on the biomass of *B.* *chinensis*. Compared with the Pb800 control, the biomass of the high DOM_CHM_ treatment significantly decreased by 42.6% for shoots and 81.8% for roots in the Pb800 soil, respectively (*p* < 0.05).

### 3.2. Pb Concentrations inShoots and Roots

The application of DOM extracts from COF, COM and CHM affected differentially (varying with DOM source and dosage, and soil Pb level) Pb concentrations in shoots and roots of *B.* *chinensis* (Table 3). Compared with the control, root Pb concentration was significantly increased in low and high DOM_CHM_ treatments at the three soil Pb levels, with statistical difference for all the DOM_CHM_ treatments except the low DOM_CHM_ treatment at Pb800 level (*p* < 0.05). For DOM_COF_ and DOM_COM_, root Pb concentration was significantly increased in the high-dosage treatments but not in the low-dosage ones at the Pb0 level compared to the control at the Pb0 level, and was not significantly different between the DOM treatments and the control at the Pb100 and Pb800 levels, expect for high DOM_COM_ dosage treatment. A significant increase in shoot Pb concentration was observed for DOM treatments at the Pb800 level compared to the control. There was no significant difference in shoot Pb concentration between other DOM treatments and the corresponding controls at the Pb0 and Pb100 soil. As expected, Pb concentrations in shoots or roots increased with increasing soil Pb level for any of the three DOM sources.

**Figure 1:**
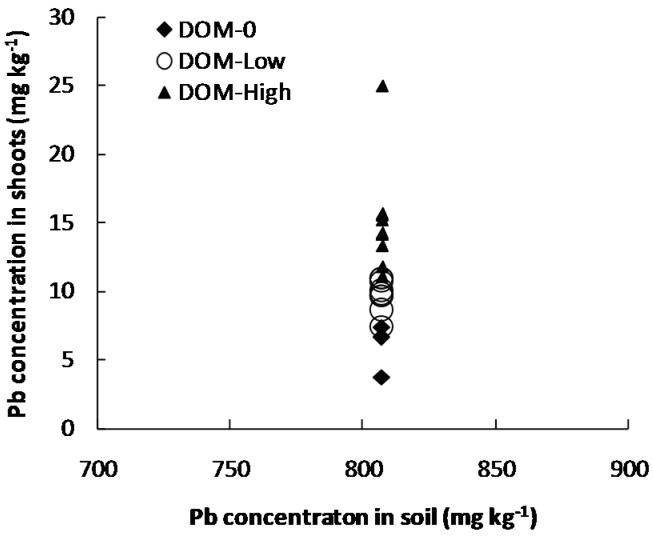
Pb concentrations in shoots of *Brassica* *chinensis* for different DOM (dissolved organic matter) levels. DOM-0, DOM-Low and DOM-High indicate DOM-free treatment, low dosage DOM treatment and high dosage DOM treatment, respectively.

Irrespective of DOM sources, Pb concentrations varied 3.70–7.42 mg·kg^−1^, 7.34–10.74 mg·kg^−1^ and 11.06–24.95 mg·kg^−1^ for DOM-free treatment, low dosage DOM and high dosage DOM, respectively, in shoots (edible parts) of *B.* *chinensis* grown in 800 mg·kg^−1^ soil (Figure 1). This indicated that high dosage DOM treatment increased Pb concentrations in edible part of *B.* *chinensis* grown in the soil with high Pb level.

### 3.3. Translocation of Pb from Soil to Shoots

Basically consistent with the shoot Pb concentrations, translocation factor of Pb was the highest in the *B.* *chinensis* grown in the Pb800 soil applied with the high DOM_CHM_ treatment, and there was no significant difference between the DOM treatment and the controls at the Pb0, P100 and Pb800 levels (Table 4). Expectedly, the translocation factor of Pb decreased sharply with the increase of soil Pb level, showing significant differences between different soil Pb levels at the same DOM source or dosage.

### 3.4. Specific Lead Uptake

**Figure 2:**
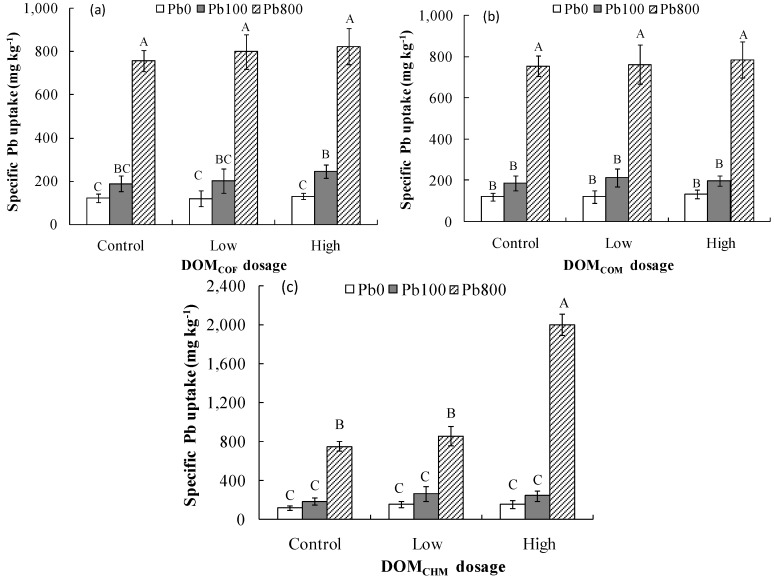
Specific lead uptake by root of *Brassica* *chinensis* under different addition dosage of DOM (dissolved organic matter) derived from: commercial organic fertilizer (**a**); cow manure (**b**); and chicken manure (**c**). The different capital letters above the bars indicated significant difference (*p* < 0.01) among Pb levels and different DOM Level based on the Least Significant Difference test. Data are means ± SE (*n* = 3).

Apparently, specific lead uptake of *B.* *chinensis* was appreciably enhanced in the DOM_CHM_ treatments at all the three soil Pb levels, with a statistically significant difference between the high DOM_CHM_ treatment and the control at the Pb800 level (*p* < 0.001) (Figure 2c). There was no significant increase in SLU for DOM_COF_ and DOM_COM_ treatments relative to the controls at the three soil Pb levels (Figure 2a,b). As expected, *SLU* displayed an increasing trend with the increase of soil Pb level for the same DOM treatment (Figure 2).

### 3.5. Potential Health Risks of Pb

The daily consumption of vegetables is estimated to be 345 and 231.5 g·person^−1^·day^−1^ for adult and children inhabitants of urban areas of Tianjin city, respectively [25,26]. In this study, 300 g·person^−1^·day^−1^ was chosen as the average amount of *B. chinensis* daily consumed by Nanjing inhabitants. According to the Nanjing Bureau of Statistics, the population of Nanjing City in 2015 was 8.24 million [27]. The estimated increase of the amount of Pb ingested by the population of Nanjing City is shown in Table 5.

It can be seen from Table 5 that the total increase in the amount of Pb ingested by the inhabitants of Nanjing City in a year due to the application of DOM could potentially be in the range of 2018–9640 kg. The degree of increase in the amount of Pb ingested depended on both the source and the amount of DOM added to the soil. The DOM_CHM_ treatment showed the greatest increase in the amounts of Pb ingested.

## 4. Discussion

The present study indicates that the effect of DOM on Pb uptake and translocation in *B.* *chinensis* is dependent on source and dosage of DOM, and Pb concentration in soil. It has been recognized that Pb solubility and mobility in soil depend on its chemical speciation and that dissolved forms and exchangeable forms of Pb in soil are readily available to plants [5]. Pb is prone to complex with DOM and the formation of organo-metallic complexes can increase the solubility and decrease the adsorption of Pb, resulting in an increase in Pb bioavailability and eventually Pb uptake by plants [5,28,29,30].

The increased Pb concentrations in roots of *B.* *chinensis* grown in the soils applied with DOM_CHM_ (Table 3) could be attributed to the formation of organo-Pb complexes between DOM_CHM_ molecules and Pb and thereby enhanced Pb bioavailability in soil. Increased root Pb concentration was also observed for high DOM_COF_ and DOM_COM_ treatments at the Pb0 level, but not at the Pb100 and Pb800 levels (Table 3). The observation can be explained by the following reasons. The original Pb in the Pb0 soil is considered to be less soluble and mobile than the lately added Pb in the Pb100 and Pb800 soils. The high DOM_COF_ and DOM_COM_ treatments could have improved the bioavailability of original Pb in the Pb0 soil by forming soluble organo-Pb complexes. By contrast, the lately-added Pb in the Pb100 and Pb800 soils remained highly available to plants and the presence of DOM_COF_ and DOM_COM_, which were much lower in TOC concentration than DOM_CHM_, had no positive effect on the already high bioavailability of Pb in the Pb100 and Pb800 soils. The TOC concentrations of these DOM samples differed greatly: 6605 mg·L^−1^ for DOM_CHM_, 13 times higher than that for DOM_COM_ and 95 times higher than that for DOM_COF_ (Table 1). The increased root Pb concentration for all the DOM_CHM_ treatments across the three soil Pb levels could be partially attributed to the much higher TOC concentration in DOM_CHM_. It has been reported that the degree of complexation between Pb and DOM increases with the increase of DOM concentration [5,12]. Inaba and Takenaka [31] showed that the amount of Cu taken up by the plants was related to the concentration of organic acid. Antoniadis and Alloway [32] also reported that an increase in DOM concentration increased metal uptake by plants. However, in the present study, the effects of DOM_COF_ and DOM_COM_ on Pb concentrations in *B. chinensis* were not significantly different despite the six-fold difference in TOC concentrations between them. The effect of DOM on bioavailability of heavy metals depends not only on quantity (concentration) but also quality (composition) of DOM [31,33,34,35]. The increased root Pb concentration for all the DOM_CHM_ treatments might also be attributable to the composition of DOM_CHM_. Further mechanistic studies at lab and greenhouse levels are required to clarify the reason for these results.

Increased shoot Pb concentration was observed in the *B.* *chinensis* grown in the Pb800 soil applied with high DOM, especially for high DOM_CHM_. Given the significantly decreased biomass of shoots and roots of *B. chinensis* and blackened roots (data not shown) in the high DOM_CHM_ treatment, the increased shoot Pb concentration might be attributable to the concentration effect of the decreased shoot biomass and/or enhanced Pb translocation to shoots due to damaged roots for the high DOM_CHM_ treatment. The significant increases in translocation factor of Pb and SLU for the high DOM_CHM_ treatment (Table 4, Figure 2) might be explained for the same reason. The decreased biomass in the high DOM_CHM_ treatment could be caused by the Pb toxicity to *B.* *chinensis*, which might be somehow enhanced after the addition of high DOM_CHM_ because DOM_CHM_ or high-level Pb alone had no adverse effect on the growth of *B.* *chinensis* as indicated in the Pb0 soil applied with high DOM_CHM_ and the Pb800 control, respectively (Table 2).

Traditionally, large quantities of organic fertilizers are applied to vegetable soil to improve soil physico-chemical properties [36,37]. For example, 90–105 t·hm^−2^·a^−1^ of organic fertilizer was applied to soil used to grow vegetables in Shenyang city, Liaoning province, China, 80% of which was chicken mature [38]. Though organic fertilizer can increase vegetable growth and production, it may also increase uptake and translocation of heavy metals in vegetables and may potentially pose a risk to public health due to the consumption of these vegetables. In the present study, the increase of the amount of Pb ingested by the population was estimated based on the present data and population of Nanjing City in 2015. Under the condition of DOM application, the annual increase of the amount of Pb ingested by the population of Nanjing City could potentially be 2018–9640 kg, because of consumption of *B.* *chinensis* grown in Pb polluted soil.

Unexpectedly, with the exception of the high DOM_CHM_ treatment in the Pb800 soil, in which *B.* *chinensis* displayed increased Pb concentration in shoots due to Pb toxicity, it is generally indicated in the present study that Pb concentration in edible shoots part of *B.* *chinensis* under normal growth was not significantly influenced by the application of DOM of various sources differing greatly in TOC concentration at different soil Pb levels, despite that Pb concentration in roots was enhanced in some DOM treatments. The result suggests that as far as DOM is concerned, the risk of Pb pollution of *B.* *chinensis* by the presence of DOM in soil originated from these organic fertilizers could be negligible. On the other hand, the application of DOM, in particular, DOM_CHM_, did increase Pb concentration in roots of *B.* *chinensis* in some treatments as indicated in the present study. The risk can rise that Pb accumulated in roots may be translocated to shoots under some conditions as seen in the high DOM_CHM_ treatment in Pb800 soil. It should be noted that the present study used only DOM extracts from some organic fertilizers, and in practice, enhanced presence of DOM occurs in soils incorporated with organic fertilizers. Further experiments at glasshouse and field levels considering practical conditions associated with organic fertilization in vegetable production are needed to understand the effect of DOM derived from various organic fertilizers on Pb uptake, translocation and pollution risk of Pb in *B.* *chinensis* and other vegetables.

## 5. Conclusions

Effect of DOM on uptake and translocation of Pb in *B. chinensis* depended on DOM sources, DOM dosage and Pb pollution level. DOM_CHM_ significantly increased Pb concentrations in roots of *B. chinensis* grown in the low and high Pb soils, while DOM_COF_ and DOM_COM_ at low dosage had no significant effect on root Pb concentration in the low Pb soil. There was no significant increase in shoot Pb concentration for all the DOM treatments except the high DOM_CHM_ treatment in the soil with 800 mg·kg^−1^ Pb. Consistent with the Pb concentrations in shoots, translocation factor of Pb from soil to shoot was higher for the high DOM_CHM_ treatment than for other DOM treatments. Apparently, the application of DOM_CHM_ as well as DOM_COF_ and DOM_COM_ at high dosage increased the SLU by *B.* *chinensis*, showing a statistically significant increase for the high DOM_CHM_ treatment in the high Pb soil. The application of DOM to the soil with 800 mg·kg^−1^ Pb could result in an increase in total Pb annually ingested by the inhabitants of Nanjing City in the range of 2018–9640 kg, with the highest estimates resulting from the high DOM_CHM_ treatment. This study suggests that as far as DOM is concerned, the risk of Pb pollution of *B.* *chinensis* by enhanced presence of DOM due to organic fertilization could be negligible. On the other hand, the risk may rise that Pb accumulated in roots could be translocated to shoots under some conditions as indicated in high DOM_CHM_ treatment and high Pb pollution level.

## Figures and Tables

**Table 1:** Physicochemical properties of dissolved organic matter (DOM) from different sources.

Source of DOM	pH	TOC mg·L^−1^	Total Pb mg·L^−1^	Total Cd mg·L^−1^	Total Cr mg·L^−1^	Total Cu mg·L^−1^	Total Zn mg·L^−1^
COF	7.21	69	nd	nd	nd	0.01	0.03
COM	7.16	482	nd	nd	nd	0.02	0.09
CHM	7.19	6605	nd	nd	0.02	0.03	2.29

nd: stands for concentration below the detection limit; DOM: represents dissolved organic matter; COF: commercial organic fertilizer; COM: cow manure; CHM: chicken manure.

**Table 2:** Dry biomass of shoots and roots of *Brassica* *chinensis* treated with varying amounts of DOM from different sources at different soil Pb levels.

DOM Source	DOM Dosage	Shoot Biomass (g)			Root Biomass (g)		
		Pb0	Pb100	Pb800	Pb0	Pb100	Pb800
control	0	3.39 ± 0.24a ^a^	3.59 ± 0.33a ^a^	3.29 ± 0.43a ^a^	0.10 ± 0.02a ^a^	0.15 ± 0.03a ^a^	0.11 ± 0.02a ^a^
COF	Low	3.60 ± 0.49a ^a^	3.84 ± 0.30a ^a^	3.31 ± 0.17a ^a^	0.13 ± 0.04a ^a^	0.12 ± 0.02a ^a^	0.08 ± 0.02a ^a^
	High	3.67 ± 0.27a ^a^	3.65 ± 0.09a ^a^	3.60 ± 0.23a ^a^	0.12 ± 0.02a ^a^	0.11 ± 0.01a ^a^	0.12 ± 0.01a ^a^
COM	Low	3.46 ± 0.13a ^a^	3.82 ± 0.42a ^a^	4.07 ± 0.69a ^a^	0.11 ± 0.01a ^a^	0.15 ± 0.04a ^a^	0.12 ± 0.03a ^a^
	High	3.61 ± 0.36a ^a^	4.06 ± 0.56a ^a^	3.54 ± 0.18a ^a^	0.12 ± 0.03a ^a^	0.18 ± 0.04a ^a^	0.11 ± 0.01a ^a^
CHM	Low	3.56 ± 0.21a ^a^	4.01 ± 1.04a ^a^	4.09 ± 0.39a ^a^	0.09 ± 0.02a ^a^	0.11 ± 0.06a ^a^	0.13 ± 0.01a ^a^
	High	3.78 ± 0.71a ^a^	4.46 ± 0.74a ^a^	1.89 ± 0.35b ^b^	0.09 ± 0.03a ^a^	0.14 ± 0.05a ^a^	0.02 ± 0.01b ^b^

DOM: stands for dissolved organic matter; COF: represents commercial organic fertilizer; COM: cow manure: CHM: chicken manure; Pb0, Pb100 and Pb800 denote the pot soils to which 0, 100 and 800 mg·kg^−1^ Pb were added, respectively. The different superscript letters in the same row indicate significant difference in shoot or root biomass (*p* < 0.05) among the soil Pb levels according to the LSD (Least Significant Difference) test. The different letters following the data in the same column indicate significant difference in shoot or root biomass (*p* < 0.05) among DOM treatments according to the LSD test. Data are means ± SE (*n* = 3).

**Table 3:** Pb concentrations in shoots and roots of *Brassica* *chinensis* treated with varying amounts of DOM from different sources at different soil Pb levels.

DOM Source	DOM Dosage	Pb Concentration in Shoots (mg·kg^−1^)			Pb Concentration in Roots (mg·kg^−1^)		
		Pb0	Pb100	Pb800	Pb0	Pb100	Pb800
control	0	4.05 ± 0.31a ^c^	6.73 ± 0.26a ^b^	7.42 ± 1.33c ^a^	2.13 ± 0.17b ^c^	24.99 ± 1.50bc ^b^	467.37 ± 23.35bc ^a^
COF	Low	4.62 ± 1.78a ^b^	6.76 ± 0.31a ^b^	10.72 ± 1.43b ^a^	2.00 ± 0.19b ^c^	25.58 ± 0.83b ^b^	442.16 ± 15.88bc ^a^
	High	3.87 ± 0.59a ^c^	8.56 ± 1.72a ^b^	13.28 ± 1.06b ^a^	8.78 ± 1.24a ^c^	23.97 ± 1.63bc ^b^	437.54 ± 27.72bc ^a^
COM	Low	3.72 ± 0.74a ^b^	7.36 ± 0.47a ^a^	9.66 ± 1.13bc ^a^	2.66 ± 0.45b ^c^	25.08 ± 0.65b ^b^	449.50 ± 29.88bc ^a^
	High	3.88 ± 0.22a ^c^	7.70 ± 1.31a ^b^	11.79 ± 1.98b ^a^	9.17 ± 1.64a ^c^	19.81 ± 1.42c ^b^	403.09 ± 20.15c ^a^
CHM	Low	3.14 ± 0.25a ^c^	7.39 ± 0.70a ^b^	10.94 ± 2.36b ^a^	8.56 ± 0.43a ^c^	37.34 ± 3.49a ^b^	507.21 ± 29.27b ^a^
	High	3.90 ± 0.15a ^c^	7.05 ± 0.71a ^b^	18.10 ± 1.72a ^a^	8.97 ± 0.72a ^c^	42.86 ± 5.58a ^b^	737.83 ± 82.58a ^a^

DOM: stands for dissolved organic matter; COF: represents commercial organic fertilizer; COM: cow manure; CHM: chicken manure; Pb0, Pb100 and Pb800 denote the pot soils to which 0, 100 and 800 mg·kg^−1^ Pb were added, respectively. The different superscript letters in the same row indicate significant difference in Pb concentrations (*p* < 0.05) among the soil Pb levels according to the LSD (Least Significant Difference) test. The different letters following the data in the same column indicate significant difference in Pb concentrations (*p* < 0.05) among DOM treatments according to the LSD test. Data are means ± SE (*n* = 3).

**Table 4:** Translocation factor of Pb from soil to shoots of *Brassica* *chinensis* (shoots/soil) treated with varying amounts of DOM from different sources at different soil Pb levels.

DOM Source	DOM Dosage	Pb Translocation Factor		
		Pb0	Pb100	Pb800
control	0	0.57 ± 0.04a ^a^	0.063 ± 0.002a ^b^	0.009 ± 0.003b ^c^
COF	Low	0.65 ± 0.25a ^a^	0.063 ± 0.003a ^b^	0.013 ± 0.002b ^c^
	High	0.54 ± 0.08a ^a^	0.080 ± 0.016a ^b^	0.016 ± 0.002ab ^c^
COM	Low	0.52 ± 0.10a ^a^	0.069 ± 0.004a ^b^	0.012 ± 0.001b ^c^
	High	0.54 ± 0.03a ^a^	0.072 ± 0.012a ^b^	0.015 ± 0.002ab ^c^
CHM	Low	0.44 ± 0.02a ^a^	0.069 ± 0.007a ^b^	0.014 ± 0.003ab ^c^
	High	0.55 ± 0.09a ^a^	0.066 ± 0.003a ^b^	0.022 ± 0.003a ^c^

DOM: stands for dissolved organic matter; COF: represents commercial organic fertilizer; COM: cow manure; CHM: chicken manure; Pb0, Pb100 and Pb800 denote the pot soils to which 0, 100 and 800 mg·kg^−1^ Pb were added, respectively. The different superscript letters in the same row indicate significant difference in Pb translocation factor (*p* < 0.05) among the soil Pb levels according to the LSD (Least Significant Difference) test. The different letters following the data in the same column indicate significant difference in Pb translocation factor (*p* < 0.05) among DOM treatments according to the LSD test. Data are means ± SE (*n* = 3).

**Table 5:** Estimated increase in the amount of Pb ingested through vegetable consumption due to the increased concentration in the edible parts of *Brassica chinensis* resulting from the addition of DOM to the soil.

Amount of Pb	COF		COM		CHM	
	Low	High	Low	High	low	High
*I_p_* (%)	44	112	30	59	47	144
*I_a_* (kg)	2974	5287	2018	3943	3174	9640

DOM: stands for dissolved organic matter; COF: represents commercial organic fertilizer; COM: cow manure; CHM: chicken manure; *I_p_* = (*C_dom_* − *C*)/*C* × 100; *C_dom_* is the Pb concentration in the edible parts of *Brassica chinensis* when DOM is added to the soil; *C* is the Pb concentration (7.42 mg·kg^−1^) in edible part of *Brassica chinensis* grown in 800 mg·kg^−1^ Pb soil without DOM added.
